# A hydrogel-based first-aid tissue adhesive with effective hemostasis and anti-bacteria for trauma emergency management

**DOI:** 10.1186/s40824-023-00392-9

**Published:** 2023-06-02

**Authors:** Dongjie Zhang, Li Mei, Yuanping Hao, Bingcheng Yi, Jilin Hu, Danyang Wang, Yaodong Zhao, Zhe Wang, Hailin Huang, Yongzhi Xu, Xuyang Deng, Cong Li, Xuewei Li, Qihui Zhou, Yun Lu

**Affiliations:** 1 Department of Gastroenterology, The Affiliated Hospital of Qingdao University, Qingdao, 266003 China; 2 Department of Stomatology, Qingdao University, Qingdao, 266021 China; 3 Department of Stomatology, Qingdao Stomatological Hospital Affiliated to Qingdao University, Qingdao, 266003 China; 4 School of Rehabilitation Sciences and Engineering, University of Health and Rehabilitation Sciences, Qingdao, 266071 China; 5 Department of Hematology, The Affiliated Hospital of Qingdao University, Qingdao, 266003 China; 6 Zhejiang Engineering Research Center for Tissue Repair Materials, Wenzhou Institute, University of Chinese Academy of Sciences, Wenzhou, 325000 Zhejiang China

**Keywords:** Tissue adhesive, Self-healing, Anti-infection, Hydrogel, Carboxymethyl chitosan, Trauma emergency

## Abstract

**Background:**

Clinical tissue adhesives remain some critical drawbacks for managing emergency injuries, such as inadequate adhesive strength and insufficient anti-infection ability. Herein, a novel, self-healing, and antibacterial carboxymethyl chitosan/polyaldehyde dextran (CMCS/PD) hydrogel is designed as the first-aid tissue adhesive for effective trauma emergency management.

**Methods:**

We examined the gel-forming time, porosity, self-healing, antibacterial properties, cytotoxicity, adhesive strength, and hemocompatibility. Liver hemorrhage, tail severance, and skin wound infection models of rats are constructed in vivo, respectively.

**Results:**

Results demonstrate that the CMCS/PD hydrogel has the rapid gel-forming (~ 5 s), good self-healing, and effective antibacterial abilities, and could adhere to tissue firmly (adhesive strength of ~ 10 kPa and burst pressure of 327.5 mmHg) with excellent hemocompatibility and cytocompatibility. This suggests the great prospect of CMCS/PD hydrogel in acting as a first-aid tissue adhesive for trauma emergency management. The CMCS/PD hydrogel is observed to not only achieve rapid hemostasis for curing liver hemorrhage and tail severance in comparison to commercial hemostatic gel (Surgiflo ®) but also exhibit superior anti-infection for treating acute skin trauma compared with clinical disinfectant gel (Prontosan ®).

**Conclusions:**

Overall, the CMCS/PD hydrogel offers a promising candidate for first-aid tissue adhesives to manage the trauma emergency. Because of the rapid gel-forming time, it could also be applied as a liquid first-aid bandage for mini-invasive surgical treatment.

**Graphical Abstract:**

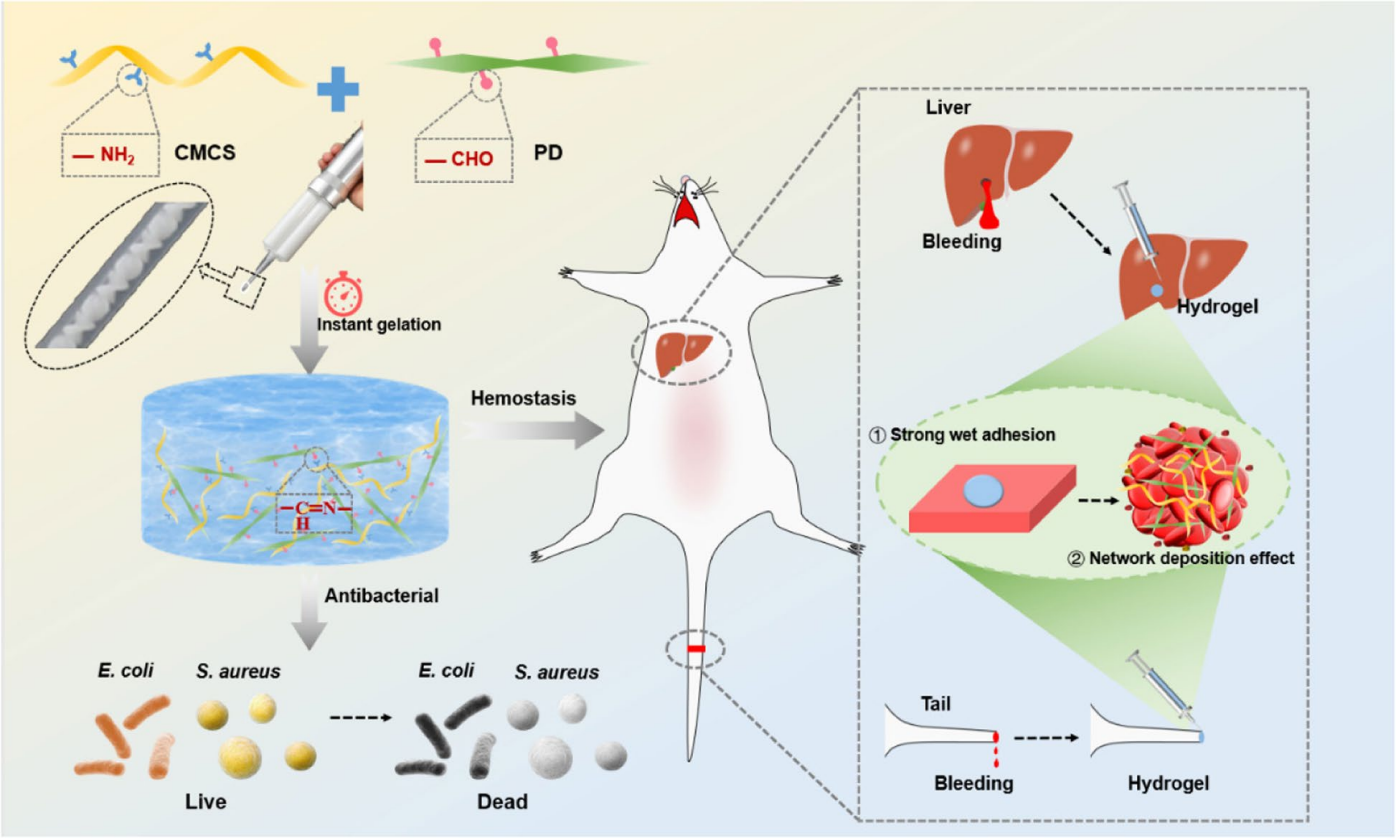

**Supplementary Information:**

The online version contains supplementary material available at 10.1186/s40824-023-00392-9.

## Introduction

Tissue injuries caused by unexpected emergencies (e.g., natural disasters and traffic accidents) are generally involved in acute hemorrhage, tissue defect, wound infection, and even tissue necrosis [1–3]. Traditionally, such emergency traumas were managed by the combined utilization of gauze and suturing/stapling. However, the conventional strategy is always accompanied by well-known drawbacks, such as the deficiency in suppressing bleeding and infection, the easy-to-cause secondary injury, and the requirement for professional staff, including surgeons and anesthetists [4]. To overcome the issues, over the past few years, tissue adhesives have gained increasing popularity as an alternative method, such as fibrin-based or cyanoacrylate-based sealants [5–7]. Unfortunately, the currently developed tissue adhesives remain constrained, describing their low adhesive strength, allergic reactions, or poor resistance to infection. Therefore, it is urgent to explore a novel first-aid tissue adhesive to satisfy the above characteristics, aiming to achieve flexible wound closure, rapid hemostasis, and effective anti-infection for trauma emergency management.

Recently, hydrogel-based tissue adhesives have gained great attention in surgical practice [8]. Among them, injectable hydrogels are deemed to possess outstanding characteristics for first-aid tissue adhesives due to their capability of filling irregularly shaped wounds and reaching the deep position of bleeding wounds [9]. As such, various materials have been applied to develop injectable hydrogels with different biofunctions, including conductivity [10], self-healing [11], and tissue adhesion [12]. Although great progress has been achieved, there is still a lack of an effective injectable hydrogel that simultaneously contains excellent biocompatibility, rapid hemostasis, strong adhesive strength, and good anti-infective property to act as the first-aid tissue adhesive for trauma emergency management. As a type of natural material, polysaccharides (e.g., dextran) offer a promising candidate for first-aid tissue adhesive because of their biodegradability, structural functionalities, as well as rapid hemostasis [13–16]. Given the concurrently promoted neovascularization of dextran for skin regeneration [17, 18], the oxidized dextran has been extensively applied to form a series of in situ hydrogels for applications in hemostatic agents, antibacterial materials, tissue adhesives, smart drug delivery vehicles, and cell carriers, because their abundant aldehyde groups could rapidly react with the amine groups of tissue/cells [19]. Chitosan, an attractive alternative to wound dressings, is a classical marine polysaccharide that contains plentiful amine groups for efficient anti-bacteria [20–23]. To improve water solubility, carboxymethyl chitosan (CMCS) was developed and demonstrated to show non-toxicity, antibiosis, non-antigenicity, and hemostasis [9, 17, 24]. In this context, we speculate that introducing CMCS into the polyaldehyde dextran (PD) is inclined to aid the formation of an injective hydrogel with excellent biocompatibility, rapid hemostasis, and anti-infection, which offers a good prospect of developing an effective first-aid wound sealant for trauma emergency management.

Based on the above description, herein, PD was synthesized by oxidizing dextran chemically with NaIO_4,_ and then the CMCS/PD hydrogels were developed based on Schiff-base reactions. Attributing to their unique physicochemical properties, the formed CMCS/PD hydrogels are speculated to exhibit the following superior merits: (1) the good injectability allows the hydrogel to accommodate various wounds with different sizes and thicknesses; (2) the accelerated gelation process could effectively seal the wound and stop the bleeding; (3) the enhanced wet-adhesive strength and self-healing capacity could achieve effective hemostasis at the wound site; (4) the good antimicrobial activity could protect wounds from contamination and infection. To verify the issues, the self-healing, tissue adhesiveness, biocompatibility, and antibacterial properties of CMCS/PD hydrogels were evaluated in vitro. After that, liver hemorrhage, tail severance, and skin infection models of rats were further constructed in vivo, to prove-of-concept and evaluate the potential of the CMCS/PD hydrogel in acting as first-aid tissue adhesives for trauma emergency management.

## Materials and methods

### Preparation and characterizations of CMCS/PD hydrogels

#### Fabrication of CMCS/PD hydrogels

CMCS (Mw: 20 kDa), a degree of deacetylation of more than 80%, was purchased from Shanghai yuan ye Bio-Technology Co. Ltd. (Shanghai, China). To synthesize PD, two solutions with different molar ratios (1:1 and 2:1) of dextran/sodium periodate (NaIO_4_) were prepared by co-dissolving dextran (2.5 mg/mL, Mw: 20 kDa, Shanghai yuan ye Bio-Technology Co. Ltd., China) and NaIO_4_ (3.3 mg/mL or 1.65 mg/mL, Shanghai yuan ye Bio-Technology Co. Ltd., China) into the double-distilled water (DDW), followed by the stirring for 24 h in the dark. After that, the solutions were dialyzed with dialysis cassettes (Mw cutoff: 8–10 kDa) against DDW for 3 d to remove the salt ions. Lastly, the resultant solutions were filtered, cast on glass dishes, and lyophilized to obtain spongy PD. The obtained PD with different theoretical oxidation degrees of 100% and 50% (corresponding to the above two couples of dextran/NaIO_4_) were designated as PD100 and PD50, respectively.

To fabricate the CMCS/PD hydrogels, CMCS solution at a concentration of 10% w/v and PD solution at a concentration of 10% w/v were prepared using phosphate-buffered saline (PBS), respectively. After that, a mixture of CMCS solution and PD solution with equal volume was performed to allow the dynamic covalent Schiff-base reaction for hydrogel formation. Gel-forming process and gelation time were recorded. Such obtained PD100- or PD50-mediated CMCS hydrogel was designated as CP100 or CP50, respectively.

#### Surface chemistry and morphology of CMCS/PD hydrogels

To analyze the surface chemistry of samples, Fourier transforms infrared (FTIR) spectroscopy of PD100, CP50, and CP100 were qualitatively analyzed by A Nicolet iN10 FTIR spectrometer (Thermo Fisher Scientific, USA) over the wavenumber range of 4000 − 500 cm^−1^. Data collection was performed by the accumulation of 32 scans with a resolution of 2 cm^−1^.

To observe the morphology of samples, CP50 and CP100 were lyophilized and the scanning electron microscope (SEM) images were performed by SEM (VEGA3, TESCAN, Czech) operated at an acceleration voltage of 10 kV. Before imaging, the hydrogel samples were Sputtercoated with gold for 60 s to increase conductivity.

#### Swelling ratios and mechanical properties of CMCS/PD hydrogels

The swelling ratio of CP50 and CP100 according to the soaking time in PBS was measured using the weighing method at 37 °C. Briefly, the initial weight of samples with constant size (1.5 cm diameter, 5 mm thickness) was measured as *M*_*0*_. Then, the samples were placed into 10 mL PBS for 400 min at 37 ℃, and the real-time swelling ratio was recorded. That is, at certain time intervals, the samples were taken out followed by the careful movement of water on the substrate surface with filter paper. Then, the hydrogel weight was measured as *M*_*s*_ and the swelling ratio (%) was evaluated as the following formula:

$$\mathrm{Swelling ratio }\left(\mathrm{\%}\right)=\frac{{M}_{s}-{M}_{0}}{{M}_{0}}$$

To analyze the mechanical properties of samples, the storage modulus (G′) and the loss modulus (G″) of hydrogels as a function of angular frequency were recorded using a rheometer (MCR 301, Anton Paar Instrument, Austria). All hydrogels (cylinder, 25 mm diameter, 1 mm height) were carried out over an angular frequency omega of 0.1–100 rad/s with a constant amplitude gamma of 1%.

### Self-healing, tissue adhesiveness, and anti-bacteria of CMCS/PD hydrogels

#### Self-healing of CMCS/PD hydrogels

To analyze the self-healing of CMCS/PD hydrogels at the macroscopic level, CP100 pre-solutions with different dyes (red and blue) were prepared and then the color-labeled CP100 hydrogels with “W” or “I” shape were formed using corresponding molds. After being cut into two pieces, the hydrogels with different colors were put together for 120 min, and the self-healing status of CP100 was recorded and observed using an optical microscope (Nikon A1 MP, Japan).

To verify the self-healing of CMCS/PD hydrogels at the microscopic level, the rheological property of CP100 (cylinder, 25 mm diameter, 1 mm height) was detected using the rotational rheometer. To determine the gel-sol transition point of the hydrogel, a strain sweep measurement was performed at the strain amplitude range of 0–500% at a fixed angular frequency (10 rad/s). Then, the self-healing property of the hydrogel was characterized by measuring G’ and G’’ under a time sweep mode at alternating low and high strain amplitudes of 1% (300 s for each interval) and 10% (200 s for each interval), respectively.

#### Tissue adhesiveness of CMCS/PD hydrogels

The tissue adhesiveness of CP50 and CP100 was investigated by lap shear and bursting pressure tests. For the lap shear test, two porcine skin tissues (1 cm × 3 cm), purchased from a local supermarket, were covered by CMCS/PD pre-solution (i.e., precursor solution, 1 cm × 1 cm) using a vortex mixer. After being contacted closely with opposite direction overlaps at room temperature for 60 min, the specimens were tested for tensile shear strength using a tabletop tensile tester (INSTRON 3382, USA) at a speed of 10 mm/min. For the bursting pressure test, the porcine skin was fixed to a measurement device equipped with a syringe pump that is filled with PBS solution. After an incision of 2 mm length was made on the porcine skin, 500 µL CMCS/PD pre-solution was injected into the incision. After 15 min at 37 °C, the bursting pressure test was performed to test the burst pressure.

#### Anti-bacteria of CMCS/PD hydrogels

The antibacterial abilities of CP100 and CP50 were examined using Gram-positive bacteria *Staphylococcus aureus* (*S. aureus*) and Gram-negative bacteria *Escherichia coli* (*E. coli*). Briefly, the pre-solution of hydrogels was filtered through a 0.22 μM membrane filter (Millex-GP, Millipore). Then 2 mL pre-solution was used to form hydrogels, followed by soaking in the sterilized PBS for 30 min. Then, 5 mL of bacterial suspension (4 × 10^9^ CFU/mL) was added to the hydrogels for co-incubation of 12 h at 37 °C in a relatively humidified atmosphere. After that, 10 µL of the treated bacterial suspension was inoculated into an agar plate for 24 h at 37 °C. Lastly, the CFUs on the agar plate were counted and the antibacterial rate was calculated as the following formula:

$$\mathrm{Antibacterial rate }\left(\mathrm{\%}\right)=\frac{{\mathrm{CFU}}_{\mathrm{s}}\mathrm{ of control}-{\mathrm{CFU}}_{\mathrm{s}}\mathrm{ of hydrogels}}{{\mathrm{CFU}}_{\mathrm{s}}\mathrm{ of control}}$$

The antibacterial activity of CMCS/PD hydrogels was also investigated using SEM. Briefly, 20 µl of the bacterial solution (4 × 10^9^ CFU/mL) was seeded on the sterilized hydrogels (height 10 mm, diameter 15 mm) and incubated for 12 h at 37 °C. Then, the hydrogels were treated with 2.5% glutaraldehyde (Shanghai yuan ye Bio-Technology Co. Ltd., China) for 12 h, followed by the dehydration of gradient ethanol (30%, 40%, 50%, 60%, 70%, 80%, 90%, 100%) for 15 min. After that, the morphology of bacteria on the CMCS/CP hydrogels was observed by SEM.

### Biocompatibility of CMCS/PD hydrogels

#### Hemocompatibility of CMCS/PD hydrogels

The hemolysis ratios (HR) of CP100 and CP50 were tested in the diluted rat blood containing 20% anticoagulated blood and 80% PBS. Briefly, 100 μL of CP100 or CP50 pre-solution was injected into 96-well plates to form hydrogels, followed by the washing of PBS three times. Then, 1 mL of the diluted blood was added to incubate the hydrogels for 1 h at 37 ℃. Meanwhile, the anticoagulated blood (0.2 mL) diluted with PBS (0.8 mL) or DDW (0.8 mL) was used as the negative or positive control, respectively. Lastly, the solutions were centrifuged at 1400 rpm for 5 min and the absorption of the supernatant in hydrogel (*OD*_*hydrogel*_), positive (*OD*_*positive*_), or negative (*OD*_*negative*_) groups was recorded at 540 nm by the microplate reader. HR was calculated according to the following formula:

$$\mathrm{HR }\left(\mathrm{\%}\right)=\frac{{OD}_{hydrogel}-{OD}_{negative}}{{OD}_{positive}-{OD}_{negative}}\times 100\%$$

To verify the hemocompatibility of CMCS/PD hydrogel, the adhesion of red blood cells (RBCs) on CP100 was observed by SEM. Briefly, the anticoagulated rat blood was incubated with CP100 for 1 h at 37 ℃. After being rinsed thoroughly with PBS to remove the unattached RBCs, the hydrogel was treated with 2.5% glutaraldehyde for 24 h, followed by the dehydration of gradient ethanol for 15 min. Finally, the morphology of RBCs on hydrogel was observed using SEM.

To examine the hemostasis of hydrogels, 400 μL of CP100 or CP50 pre-solution was mixed with 100 μL rat blood in a 1.5 mL glass bottle. 100 μL of pure rat blood was set as the control group. After stranding for 5 s, 1 mL DDW was added to incubate each sample for 10 min, aiming to hemolyze the non-trapped RBCs to release hemoglobin into the water [25]. Lastly, the hemoglobin concentration was determined by measuring the absorbance at 545 nm using the microplate reader.

#### Cytocompatibility of CMCS/PD hydrogels

The hydrogel-extracted solution was used to analyze the effect of CP100 and CP50 on the behavior of mouse L929 fibroblast cells (Shanghai YanJin Biotech Co. Ltd., China). Briefly, the CMCS and PD solutions were exposed to ultraviolet radiation for 30 min and then the hydrogel was formed in a sterile operating environment. Hydrogels formed from 100 μL pre-solution were immersed into 8 mL DMEM (Biological Industries, Israel) for 12 h at 37 ℃. Then, the treated medium was collected and filtered through a 0.22 μM membrane filter. The obtained solution was set as the hydrogel-extracted solution. To observe the cell proliferation, L929 cells were seeded in 96-well plates at a density of 5 × 10^3^ cells/well and cultured with DMEM medium for 24 h. Then, the hydrogel-extracted solution was used to culture cells for a further 1, 3, and 5 days. After that, the samples were collected and incubated with 100 μL of fresh DMEM containing 10% Cell Counting Kit 8 (CCK-8, Dojindo Molecular Technologies, Japan) solution for 1 h at 37℃ and the absorbance of the solution at 450 nm was performed using a microplate reader. To evaluate the cell cytotoxicity, L929 cells were seeded in 6-well plates at a density of 3 × 10^4^/well for 12 h. Then, the hydrogel-extracted solution was used to culture cells for a further 24 h. After that, Live/Dead fluorescent staining was performed using 2.5 μM calcein-AM and 2.5 μM ethidium homodimer (Dalian Meilun Bio-Technology Co. Ltd., China) solution for 15 min of incubation. Lastly, fluorescence images were taken under an inverted fluorescence microscope (Nikon A1 MP, Japan) and the cell survival rate (the percentage of living cell number in all cells) was calculated.

### In vivo animal test of CMCS/PD hydrogels as first-aid tissue adhesives

#### In vivo hemostatic ability

Hemorrhage models of rat liver and tail were built to simulate the human visceral and dismembered hemorrhage, respectively. Briefly, 8-week-old male SD rats (200-220 g) were heparinized with intravenous use of 2.5 U/g heparin [26]. After successful anesthetization of rats by intraperitoneal injection with 1% w/v pentobarbital solution, the liver was carefully exposed and placed on pre-weighed filter paper, followed by an incision with a length of 3 mm formed on the liver surface using a surgical blade. Similarly, the broken tail model was established by cutting off the rat tail. After that, the CP100 pre-solution was immediately sprayed onto the bleeding sites using a vortex mixer, and the hemostatic status was recorded based on the calculated blood loss and hemostasis time. The heparinized rats without any hemostatic interventions were set as the “control H” group, while the normal rats without any hemostatic interventions were set as the control group, and the heparinized rats treated by ﻿Surgiflo ® (Johnson & Johnson, USA), a commercial hemostatic product, was set as the positive control. 3 SD rats in each group, and 15 SD rats in each experiment.

#### In vivo antimicrobial ability

For in vivo antibacterial test, 8-week-old male SD rats (200-220 g) were anesthetized by intraperitoneal injection with 1% w/v pentobarbital solution. Then, the trauma with a diameter of 10 mm was created on the surface of the rat skin, followed by the smearing of 100 μL *S. aureus* suspension liquid (1 × 10^8^ CFU/mL). After the establishment of the skin infection model for 1 d, 200 μL of CP100 or CP50 pre-solution was injected to in situ form the hydrogels on the wounds. Commercial Prontosan ® wound gel was utilized to serve as the control group. After the procedure, the rats were individually caged with free movement and food access. Next, the infection site was photographed to trace the wound infection progression at 0, 1, and 4 d. To quantitatively analyze the infection status, the infected wound tissue at 4 d was collected and homogenized with saline. Then, the homogenate was inoculated on agar plates for 24 h at 37 ℃, and the colony number was counted. To analyze the infection-activated inflammatory response, hematoxylin and eosin (H&E, Solarbio, China) staining of the wound tissue was performed based on the full-thickness skin-containing wound (1.5 cm) according to product instructions. Images were obtained under the microscope. 3 SD rats in each group, and 12 SD rats in each experiment.

### Statistical analysis

Statistical analysis was carried out using GraphPad Prism 8.0 software. One-way analysis of variance with Tukey's test was used to determine differences between groups. A value of *p* < 0.05 was considered to be statistically significant and in the quantitative image *, **, and *** were considered to *p* < 0.05, *p* < 0.01, and *p* < 0.001, respectively. The results were expressed as the mean ± standard deviation (SD).

## Results

### Successful synthesis of CMCS/PD hydrogels

**Fig. 1:**
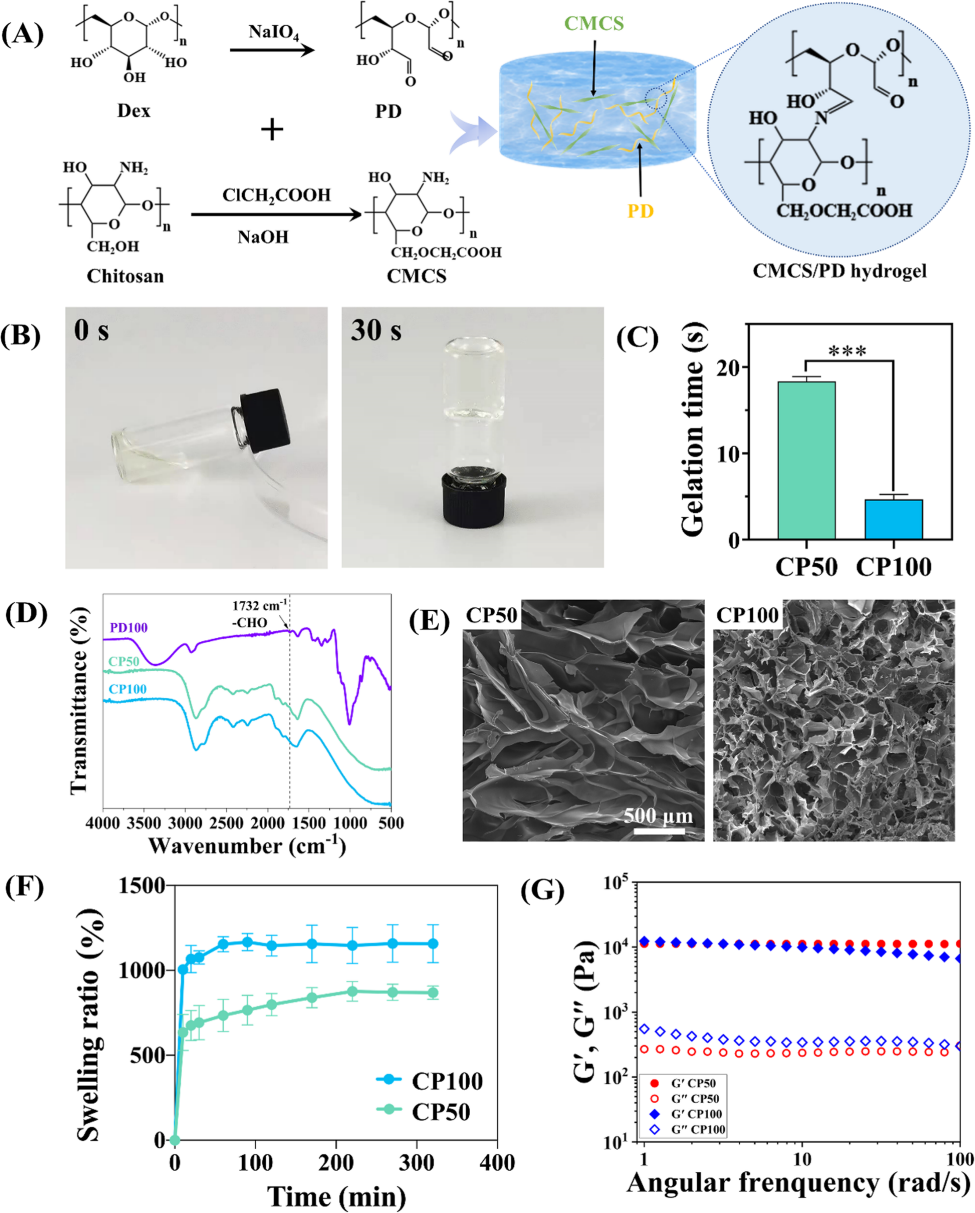
**A** Synthesis schematics of PD molecules, CMCS molecules, and CMCS/PD hydrogels. **B** Optimal images of Sol (left)-to-Gel (right) transition of CP50. **C** Gelation time of CMCS/PD hydrogels. **D** FTIR spectra of PD100, CP50, and CP100. **E**–**G** SEM images (100 ×), swelling ratio, and rheological properties of CP50 and CP100

Considering that the aldehyde groups could rapidly react with the amine groups via the dynamic Schiff-base reaction, combining PD with CMCS was speculated to be capable of developing an injective hydrogel with rapid gel-forming and self-healing abilities (Fig. 1A), thus could act as the first-aid wound sealant for effective hemostasis and anti-infection. To verify it, the oxidized dextran with aldehyde groups was synthesized firstly by the chemical treatment of NaIO_4_ in this study (Fig. S1A). The aldehyde content of PD50 and PD100, detected by hydroxylamine hydrochloride titration method [27], was 24.77 ± 1.43% and 55.25 ± 1.57%, respectively (Fig. S1C). Then, the CMCS/PD hydrogels were rapidly formed by directly mixing PD and CMCS solutions (Fig. 1B). As shown in Fig. 1C, the short gelation time confirms the rapid gel-forming property of CMCS/PD systems, which is well suitable for the development of rapid hemostatic hydrogels (Fig. S2). The increased aldehyde groups were found to significantly reduce the gelation time of CMCS/PD systems to 5 s. The chemical features characterized by FTIR reconfirmed the successful synthesis of CMCS/PD hydrogels (Fig. 1D), as the carbonyl stretching peak (1732 cm^−1^) from an aldehyde group disappeared in CMCS/PD hydrogels.

For the homogeneous porous microstructure of CMCS/PD hydrogels, the increased content of the aldehyde groups led to more Schiff-base reactions, resulting in smaller pore size and denser structure in CP100 than in CP50 (Fig. 1E). Attributing to the higher amount of hydrophilic functional groups, the swelling rate of CP100 was also higher than that of CP50 (Fig. 1F). For the mechanical properties of hydrogels, a slight difference in G′ and G″ was found between CP50 and CP100 (Fig. 1G).

### CMCS/PD hydrogels exhibit excellent self-healing and tissue adhesiveness

**Fig. 2:**
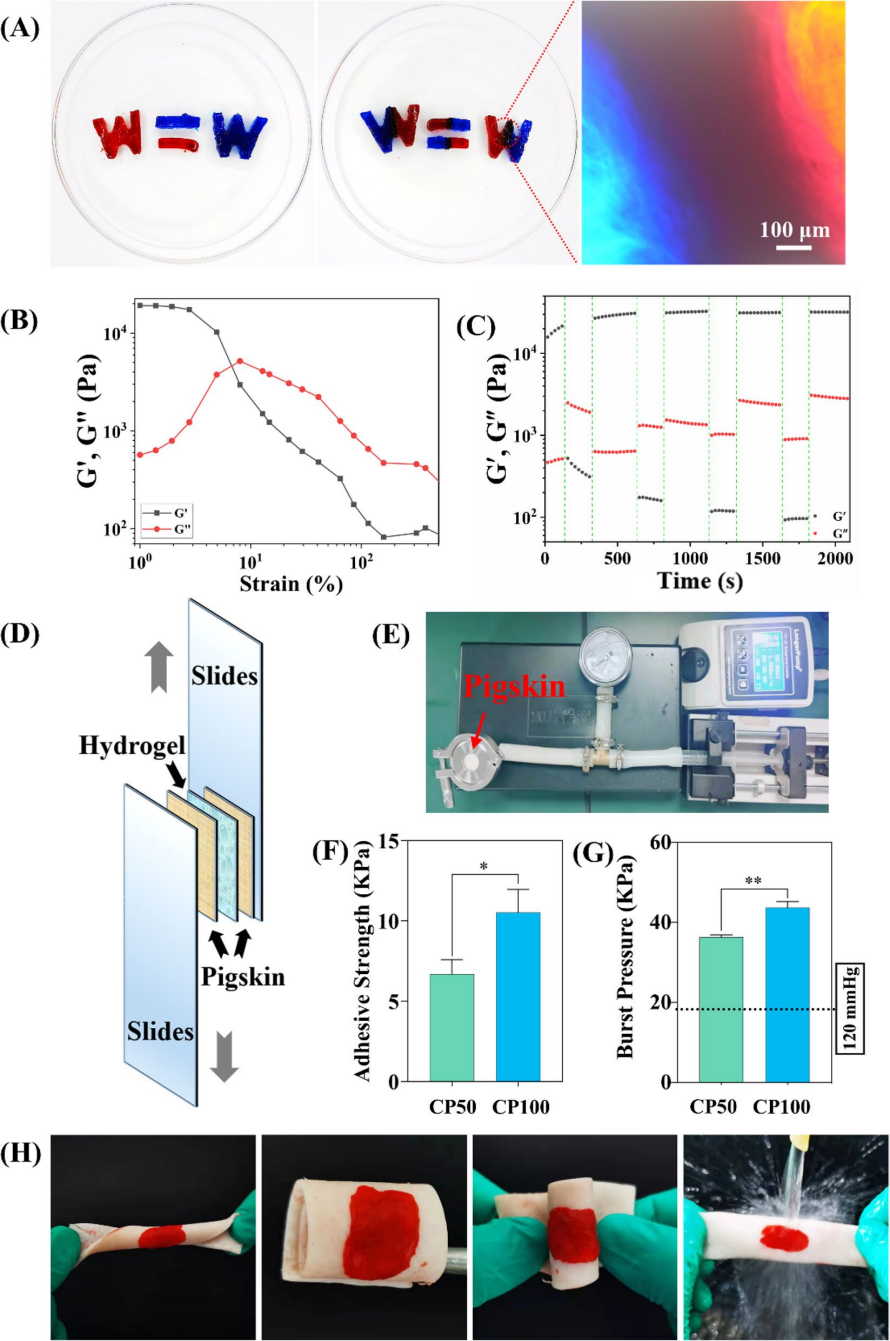
**A** Self-healing properties of CP100. **B** Rheological property of CP100 at the strain amplitude range of 0–1000% at a fixed angular frequency (10 rad/s). **C** Rheological property of CP100 under a time sweeps mode at alternating low and high strain amplitudes of 1% and 10%. **D**-**E** Schematics of lap shear and burst pressure tests. **F**-**G** Adhesive strength and burst pressure of hydrogels. **H** Photographs of hydrogels adhered to pigskin with various twisting and folding

To analyze the self-healing ability of CP100, the cut hydrogels with different colors were spliced together for 120 min. The excellent self-healing status was observed at the spliced site of hydrogels (Fig. 2A), and the formed hydrogels could effectively maintain their integrity under gravity without any external intervention (Fig. S3). This is mainly attributed to the dynamic Schiff-base reactions. To confirm it, the gel-sol transition point of the CP100 was detected using strain sweep measurement (Fig. 2B), and the rheological recovery test of CP100 was performed under a time sweep mode at alternating low and high strain amplitudes of 1% and 10% (Fig. 2C). It was found that G' was higher than G'' at 1.0% strain while lower at 10.0% strain. Nevertheless, when the strain recovered from 10.0% to 1.0%, G' could immediately recover to a high level, reconfirming the excellent self-healing property of CP100.

Lap shear and burst pressure tests were performed to detect the tissue adhesiveness of CMCS/PD hydrogels (Fig. 2D**/**E). The higher adhesive strength was found in CP100 than that in CP50 (Fig. 2F), because of the higher amount of aldehyde groups that supply more anchors for grafting the amine groups in tissue. Similarly, the average burst pressure of CP100 (327.5 ± 9.3 mmHg) was also higher than that of CP50 (272.5 ± 3.5 mmHg) (Fig. 2G), and the formed CP100 could adhere firmly to the surface of the pigskin (Fig. 2H). Furthermore, CP100 showed the obvious electrical conductivity that provides prospects for application in bio-signal detection and healthcare monitoring (Fig. S4).

### CMCS/PD hydrogels have excellent antibacterial capacity

**Fig. 3:**
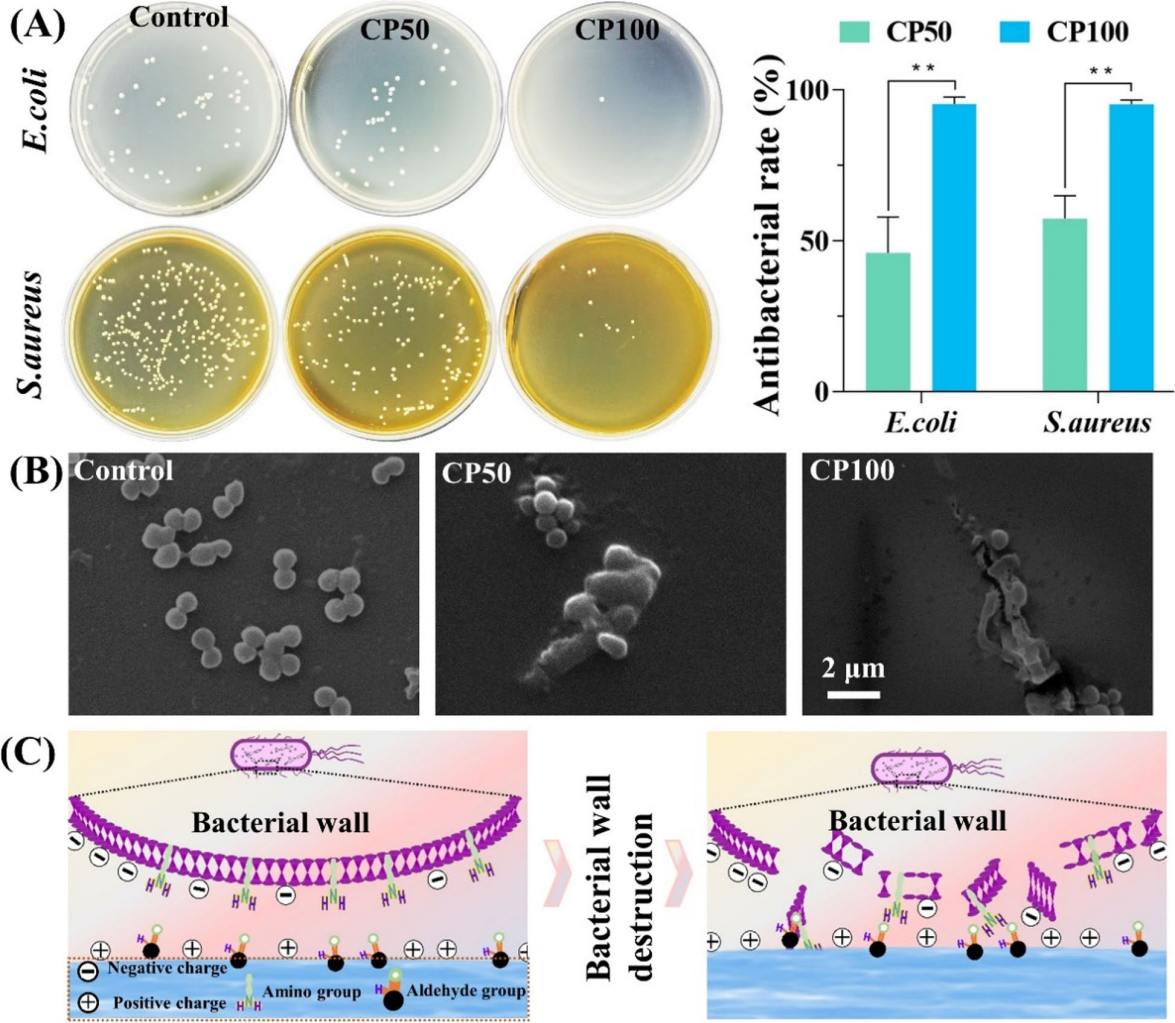
**A** Photographs of survival bacteria colonies treated by CMCS/PD hydrogels and the associated antibacterial rate calculated from image (**A**). **B** SEM images of *S. aureus* on the CMCS/PD hydrogels. **C** Antibacterial mechanism of CMCS/PD hydrogels

A significant decrease in bacterial colony number was found on CP100 compared with that of the control and CP50 groups (Fig. 3A), indicating its excellent antibacterial capacity. Concretely, the antibacterial rates of CP100 were both higher than 95% against *S. aureus* and *E. coli*. However, the antibacterial rates of CP50 were only 27.33 ± 6.03% against *E. coli* and 57.53 ± 7.51% against *S. aureus*. SEM verified the excellent antibacterial effect of CMCS/PD hydrogels via damaging the bacteria membrane (Fig. 3B**/**C).

### CMCS/PD hydrogels exhibit excellent hemocompatibility and cytocompatibility

**Fig. 4:**
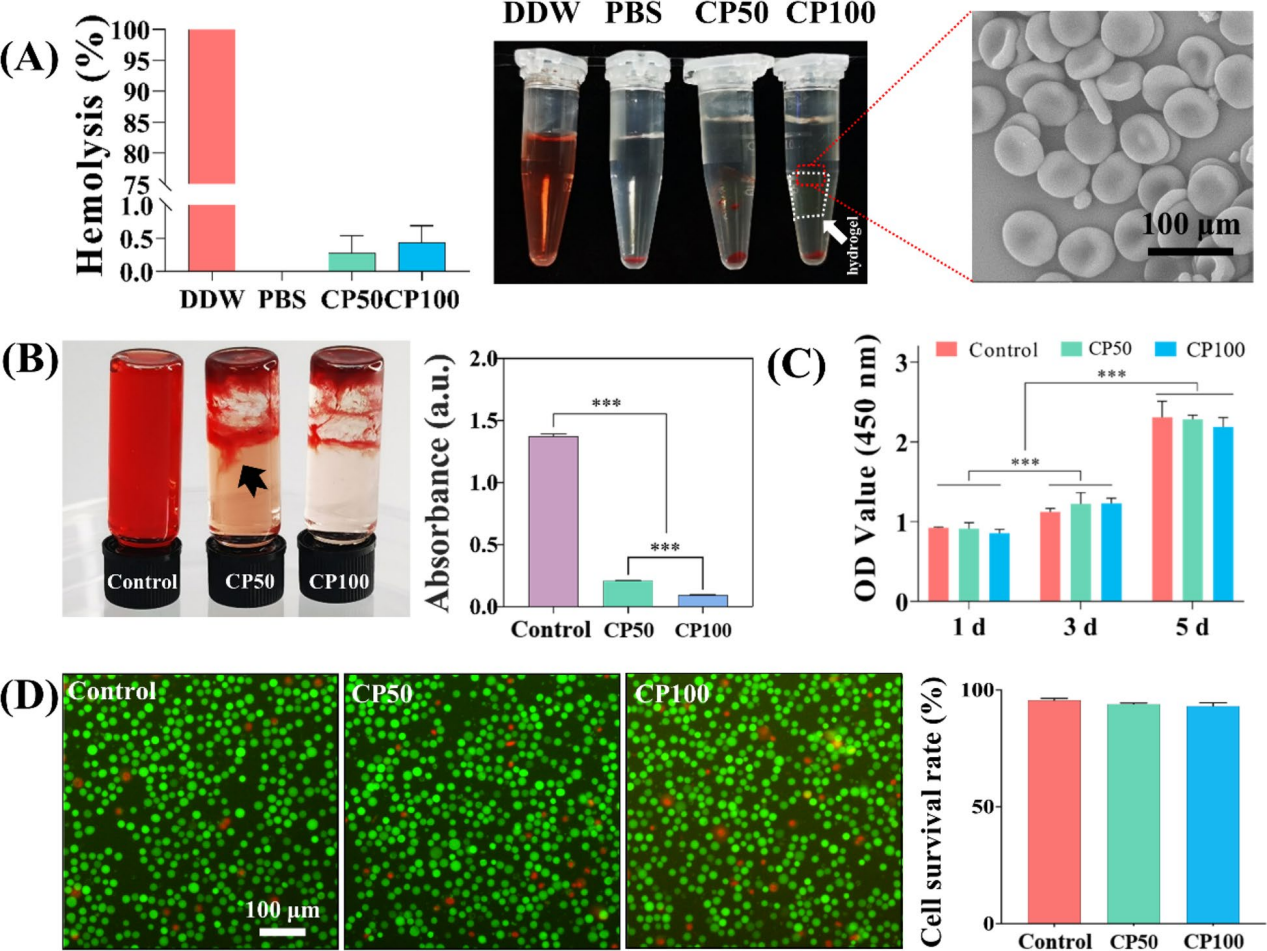
**A** Hemolysis of hydrogels and the morphology of the adhered RBCs on CP100. **B** Hemostasis of hydrogels and the quantitative analysis. The black arrow indicates blood leakage. (C) Cell proliferation. **D** Live/Dead fluorescent images and the quantitative cell survival rate

The hemolysis rates of CP100 and CP50 are both lower than 2% (Fig. 4A). For the hemostatic test, slight blood leakage was observed in the CP50 group compared with the control group (Fig. 4B, black arrow), and the detected OD value of CP100 was significantly lower than that of CP50. The RBCs adhered to the surface of CP100 hydrogel still maintained the typical biconcave disk shape.

CCK-8 and Live/Dead staining assays were performed to detect the cytocompatibility of CMCS/PD hydrogels. For cell proliferation, positive cell growth without significant difference in control, CP50 and CP100 groups, as the culture time increased from 1 to 5 d (Fig. 4C). For Live/Dead staining, the percentage of living cells was detected without significant difference among the three groups after 24 h co-incubation (Fig. 4D).

### CMCS/PD hydrogels acting as tissue adhesives achieve rapid hemostasis

**Fig. 5:**
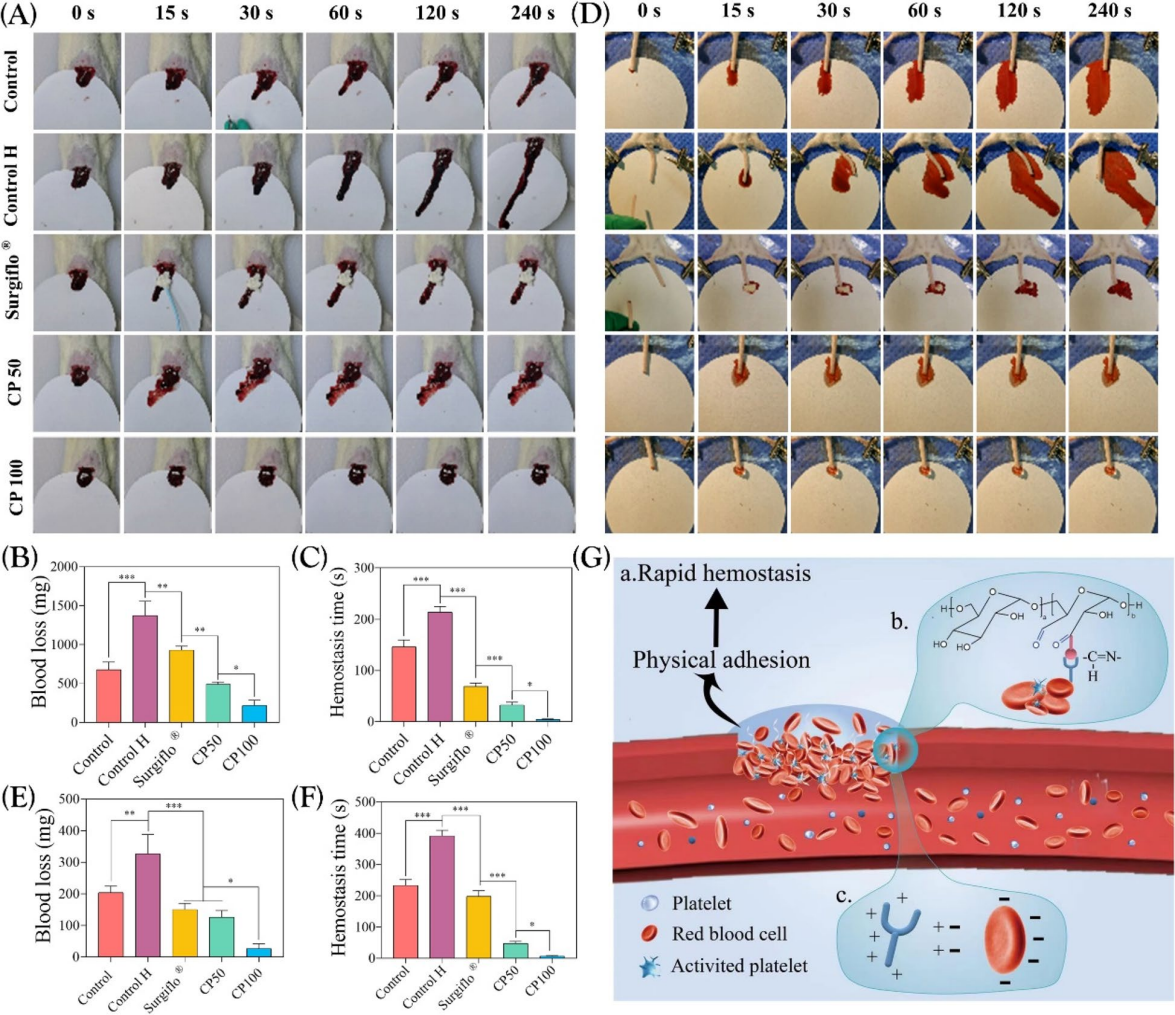
**A** Optimal images to observe the hemostatic effect of CMCS/PD hydrogels on rat liver bleeding model. **B**-**C** The quantitative blood loss and hemostasis time from image (**A**). **D** Optimal images to observe the hemostatic effect of CMCS/PD hydrogels on rat tail severance model. **E**–**F** The quantitative blood loss and hemostasis time from image (**D**). **G** Schematic diagram of hemostasis mechanisms of CMCS/PD hydrogels

First, the hemostatic property of CMCS/PD hydrogels was evaluated using a rat liver model, a specific visceral organ with abundant blood supply. In the bleeding model of rat liver, the notable blood flow on the filter paper in the Control H group confirmed the successful disruption of the rat coagulation process by heparin (Fig. 5A). Although the liver bleeding was effectively inhibited by Surgiflo ®, the hemostatic effect was still lower than that of CP100, which was confirmed by the quantitative blood loss (Fig. 5B). For hemostasis time, compared with the Control H group (213.67 ± 11.24 s), the hemostasis time of Surgiflo ®, CP50 and CP100 group was 69 ± 6.56 s, 32.67 ± 5.51 s, and 4.33 ± 1.15 s, respectively (Fig. 5C).

The rat tail severance model was also established. As shown in Fig. 5D, Surgiflo ® significantly inhibited the bleeding of the tail severance site via concentrating the blood, and CP100 achieved a rapid hemostatic effect compared to other groups (Fig. 5E**/**F).

Given the blood pressure of rabbits similar to humans, the liver and cardiac hemostasis test was also performed in rabbits, and the results further clarified the excellent hemostatic effect of CMCS/PD hydrogels (Fig. S5 and Fig. S-video1/2).

### CMCS/PD hydrogels exhibit superior anti-infection for treating skin trauma

**Fig. 6:**
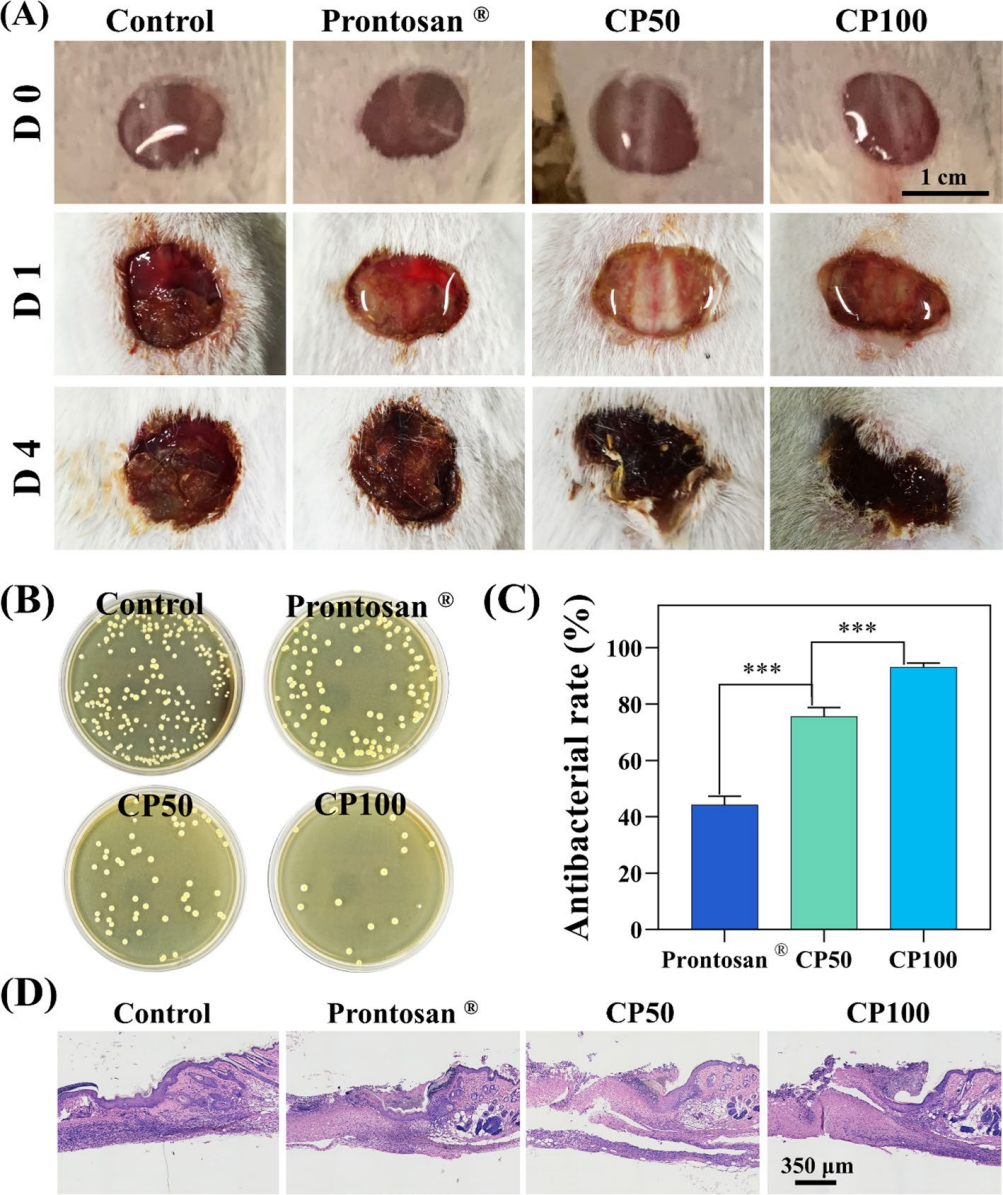
**A** Optimal images of wound infection treated by CMCS/PD hydrogels. **B** S. aureus colonies on TSB agar plates from the homogenate of the wounds in PBS. **C** Quantified colony number of *S. aureus* in the skin scab. **D** H&E staining images of surrounding wounds

*S. aureus*-induced skin infection in a rat model was used to elaborate on the potential of CMCS/PD hydrogels in treating acute skin trauma. At day 4, the wounds were found to be crusted in hydrogel and Prontosan ® groups, while the control group was prone to breakage **(**Fig. 6A**)**. Through quantifying the bacterial colonies at the wound site, a significant reduction of colony number was found in hydrogel groups in comparison to control and Prontosan ® groups **(**Fig. 6B**/**C**)**. Meanwhile, the antibacterial effect (93 ± 1.37%) of CP100 was significantly higher than that of CP50 (75.68 ± 3.13%). As such, H&E staining was implemented to assess the inflammatory infiltration in the repair process of infective skin. As shown in Fig. 6D, an obvious inflammatory response was observed in all groups.

## Discussion

Ideal first-aid tissue adhesives should possess the following characteristics: rapid hemostasis, excellent antimicrobial properties, the ability to conform to complex wound shapes, and the capacity to prevent secondary injuries [28–33]. Combining PD with CMCS through dynamic Schiff-base reactions could result in an injectable hydrogel with rapid gel-forming and self-healing capabilities, making it a promising candidate for use as a first-aid wound sealant to effectively achieve hemostasis and prevent infection. The rapid gel-forming property of CMCS/PD systems is well-appropriate for the requirement of rapid hemostatic hydrogels. Moreover, due to its high liquid absorption, CP100 facilitated blood component aggregation and clot formation, accelerating the hemostatic process. The high G' values, as compared to G'' over the range of angular frequencies (0.1–100 rad/s), indicate a stable network structure and excellent gel properties of CMCS/PD hydrogels, with G' closely associated with the connectivity of the polymeric network. Specific wound sites (e.g., elbow, hip, and knee) generally suffer from external stress during frequent movement and stretching, which easily damages the structure of tissue adhesives and causes wound infection [2, 29]. Good self-healing of materials could effectively avoid the above issues. The excellent self-healing status observed at the spliced site of CMCS/PD hydrogels is mainly attributed to the dynamic Schiff-base reactions. The excellent self-healing ability of CMCS/PD hydrogels could maintain their integrity in the aforementioned complex environments, achieving a physical barrier effect. Since the high liquid absorption is beneficial to blood component aggregation and clot formation [30], CP100 was speculated to be inclined to accelerate the hemostatic process. All the evidence demonstrated the good tissue adhesiveness of CP100, which is competent to seal the bleeding site in a harsh environment [28].

The skin acts as a protective barrier against chemical damage, mechanical injury, or microbial invasion [34]. However, once the skin is damaged, bacteria could invade the wound site, which may result in bacterial infection, sepsis, and even death [35]. The in vitro results of the antibacterial experiments indicate that the CMCS/PD hydrogels possess excellent antibacterial properties. Two mechanisms could account for the antibacterial effects of CMCS/PD hydrogels: (i) the aldehyde groups of CMCS/PD hydrogels react with the primary amine groups on the bacterial membrane, thereby inhibiting bacterial proliferation [36–38]; (ii) the cationic amine groups of CMCS react with the negatively charged bacteria, which penetrates the bacterial membrane and causes the leakage of intracellular fluids [39, 40].

A higher hemolysis ratio indicates more damage to erythrocyte cells caused by blood-contacting biomaterials. As the accepted threshold value for blood-contacting materials is 2% [41], the hemolysis rates of CP100 and CP50 are both lower than 2%, indicating the excellent hemocompatibility of CMCS/PD hydrogels. The adhered RBCs on CP100 maintained a typical biconcave disk shape without obvious cell rupture, which further confirmed its good hemocompatibility. These results demonstrate the potential of CP100 to seal vascular breaches with excellent hemocompatibility and rapid hemostasis. The CCK-8 and Live/Dead staining assays were conducted to further confirm the excellent cytocompatibility of CMCS/PD hydrogels.

Achieving rapid hemostasis remains a challenge for patients with massive bleeding or anticoagulated trauma. To evaluate the hemostatic properties of CMCS/PD hydrogels, a rat liver model was used, which is a specific visceral organ with abundant blood supply. In the bleeding model of the rat liver and tail, although Surgiflo ® effectively inhibited bleeding, the hemostatic effect was still lower than that of CP100, which was confirmed by quantitative blood loss. These results indicate that CP100 could act as a first-aid tissue adhesive for in-situ rapid hemostasis. Several underlying hemostatic mechanisms of CMCS/PD hydrogels could be summarized as follows (Fig. 5G): (a) the rapid gelling capacity and strong tissue adhesiveness effectively seal the wound and act as a physical barrier immediately [42, 43]; (b) the aldehyde groups of PD promote the adhesion and aggregation of RBCs, platelets, fibrin, and other coagulation factors from the blood via Schiff-base effects, thereby activating the coagulation cascade reaction to rapidly form clots; (c) the positively charged characteristics of CMCS molecules accelerate the aggregation of RBCs and coagulation factors for clot formation via electrostatic effects [26, 44, 45].

To elaborate on the potential of CMCS/PD hydrogels in treating acute skin trauma, a rat model of *S.aureus*-induced skin infection was used. The experiment demonstrated the superior anti-infective properties of CP100 for treating acute skin trauma. Additionally, the aggregation and activation of inflammatory cells play a critical role in the early phase of infected tissues [43, 46] and could influence the conversion between inflammatory and repair stages [47]. By relying on their chemical, physical, and biological properties, CMCS/PD hydrogels could affect wound microenvironments at the molecular level [48]. Compared with the control group, a significant reduction of inflammatory cells was found in the CP100 hydrogels, indicating the good capacity of CMCS/PD hydrogels in alleviating infection-triggered excessive inflammation. Regarding the underlying mechanisms involved, the following explanations could be given: (1) CMCS/PD hydrogels have the ability to destroy bacterial membranes and reduce the number of resident bacteria, thereby alleviating inflammation [39, 40]; (2) the hydrogel could act as a dense physical barrier to prevent further invasion of external bacteria [49]; (3) the capacity of hydrogels to absorb wound exudate could reduce inflammation for wound healing [50]. Taken together, CMCS/PD hydrogels provide an alternative strategy for the treatment of bacteria-infected wounds.

## Conclusions

A novel, self-healing, and antibacterial CMCS/PD hydrogel was successfully designed as the first-aid tissue adhesive via the dynamic Schiff-base reaction for trauma emergency management. The developed CMCS/PD hydrogel was confirmed to have rapid gel-forming and self-healing abilities for forming injective hydrogels. It also exhibits excellent anti-bacterial properties and strong tissue adhesiveness with good biocompatibility. In vivo, the CMCS/PD hydrogel not only effectively achieved rapid hemostasis for curing liver hemorrhage and tail severance via sealing wound site and mediating clot formation, but also significantly inhibited the bacteria-induced infection for treating acute skin trauma. Summarily, such designed CMCS/PD hydrogel shows prospects for wide application in first-aid tissue adhesives to manage emergency trauma.

## Supplementary Information

**Additional file 1.** The hemostatic effect of CP100 in rabbit hearts.

**Additional file 2.** The hemostatic effect of CP100 in rabbit hearts.

**Additional file 3:** **Figure S1. **(A) ^1^H NMR spectrometer (Bruker Advance III, German) of PD0, PD50, and PD100. Compared to Dex, hemiacetal peaks at 4.0–5.6 ppm appeared in CP50 and CP100 spectra, indicating that hemiacetal groups were generated in the prepared polymer backbone. (B)Fourier transforms infrared (FTIR) spectroscopy of Dex, PD50, and PD100. The FTIR spectra of PD50 and PD100 presented a weak peak at 1732 cm-1 belonging to the stretching of the carbonyl from an aldehyde group, confirming the successful oxidation of dextran residues. (C) The aldehyde content of the dextran was evaluated by the hydroxylamine hydrochloride titration method (*n*=3). The aldehyde conversion rates of PD50 and PD100 were 24.77 ± 1.43% and 55.25 ± 1.57%, respectively. (D) After 5 minutes, all the CMCS/PD hydrogels showed an similar increase in surface area growth ratio (2.69%). Until to 80 min, a significant change in surface area growth ratio was observed, in which the area growth ratio of CP100 is 8.86% whereas that of CP50 was 6.78%. **Figure S2.** Signature by pen (A) and CP100 hydrogels (B). The macroscopic injectability of the hydrogels is shown below, which proved that the CP100 hydrogels displayed favorable injectability and rapid gelation ability. Which is well suitable for the development of injective hydrogels. **Figure S3.** Self-healing effect of CP100. The excellent self-healing status was observed at the spliced site of hydrogels, and the formed hydrogels could effectively maintain their integrity under gravity without any external intervention. **Figure S4.** The electrical conductivity of DDW, PD50, PD100, CMCS, CP50 and CP100. CP100 showed the obvious electrical conductivity that provides prospects for application in bio-signal detection and healthcare monitoring. **Figure S5. **(A) Optimal images to observe the hemostatic effect of CMCS/PD hydrogels on rabbit liver bleeding model. (B-C) The quantitative blood loss and hemostasis time from image (A). Given the blood pressure of rabbits similar to humans, the cardiac hemostasis test was also performed in rabbits, and the results further clarified the excellent hemostatic effect of CMCS/PD hydrogels.

## Data Availability

Please contact the author for data requests.

## Abbreviations

CMCS: Carboxymethyl chitosan
PD: Polyaldehyde dextran
NaIO_4_: Sodium periodate
DDW: Double-distilled water
PBS: Phosphate-buffered saline
