# Supplementary material for: A hydrogel-based first-aid tissue adhesive with effective hemostasis and anti-bacteria for trauma emergency management
Source: Biomater Res. 2023 Jun 2;27:56. doi: 10.1186/s40824-023-00392-9 (PMC10236594; doi:10.1186/s40824-023-00392-9)
Supplement: Supplementary file 3 — Additional file 3: Figure S1. (A) 1H NMR spectrometer (Bruker Advance III, German) of PD0, PD50, and PD100. Compared to Dex, hemiacetal peaks at 4.0–5.6 ppm appeared in CP50 and CP100 spectra, indicating that hemiacetal groups were generated in the prepared polymer backbone. (B)Fourier transforms infrared (FTIR) spectroscopy of Dex, PD50, and PD100. The FTIR spectra of PD50 and PD100 presented a weak peak at 1732 cm-1 belonging to the stretching of the carbonyl from an aldehyde group, confirming the successful oxidation of dextran residues. (C) The aldehyde content of the dextran was evaluated by the hydroxylamine hydrochloride titration method (n=3). The aldehyde conversion rates of PD50 and PD100 were 24.77 ± 1.43% and 55.25 ± 1.57%, respectively. (D) After 5 minutes, all the CMCS/PD hydrogels showed an similar increase in surface area growth ratio (2.69%). Until to 80 min, a significant change in surface area growth ratio was observed, in which the area growth ratio of CP100 is 8.86% whereas that of CP50 was 6.78%. Figure S2. Signature by pen (A) and CP100 hydrogels (B). The macroscopic injectability of the hydrogels is shown below, which proved that the CP100 hydrogels displayed favorable injectability and rapid gelation ability. Which is well suitable for the development of injective hydrogels. Figure S3. Self-healing effect of CP100. The excellent self-healing status was observed at the spliced site of hydrogels, and the formed hydrogels could effectively maintain their integrity under gravity without any external intervention. Figure S4. The electrical conductivity of DDW, PD50, PD100, CMCS, CP50 and CP100. CP100 showed the obvious electrical conductivity that provides prospects for application in bio-signal detection and healthcare monitoring. Figure S5. (A) Optimal images to observe the hemostatic effect of CMCS/PD hydrogels on rabbit liver bleeding model. (B-C) The quantitative blood loss and hemostasis time from image (A). Given the blood pressure of rabb [file 40824_2023_392_MOESM3_ESM.docx]

**A Hydrogel-based** **First-Aid** **Tissue Adhesive with Effective Hemostasis and Anti-bacteria for** **Trauma Emergency Management**

Dongjie Zhang ^a^, Li Mei ^b^, Yuanping Hao ^c^, Bingcheng Yi ^d^, Jilin Hu ^a^, Danyang Wang ^b^, Yaodong Zhao ^a,b^, Zhe Wang ^b^, Hailin Huang ^b^, Yongzhi Xu ^c^, Xuyang Deng ^b^, Cong Li ^b^, Xuewei Li ^e^, Qihui Zhou ^d,f^* and Yun Lu ^a^*

**Author Affiliations:**

^a^ Department of Gastroenterology, The Affiliated Hospital of Qingdao University, Qingdao 266003, China.

^b^ Department of Stomatology, Qingdao University, Qingdao 266021, China.

^c^ Department of Stomatology, Qingdao Stomatological Hospital Affiliated to Qingdao University, Qingdao 266003, China.

^d^ School of Rehabilitation Sciences and Engineering, University of Health and Rehabilitation Sciences, Qingdao 266071, China.

^e^ Department of Hematology, The Affiliated Hospital of Qingdao University, Qingdao 266003, China.

^f^ Zhejiang Engineering Research Center for Tissue Repair Materials, Wenzhou Institute, University of Chinese Academy of Sciences, Wenzhou, Zhejiang 325000, China.

*Corresponding Authors

Yun Lu**­**, Ph.D., Professor of Gastroenterology, Phone: +86-18661802231, Email: [luyun@qdu.edu.cn](mailto:luyun@qdu.edu.cn)

Qihui Zhou, Ph.D., Professor of Biomaterials, Phone: +86-17660670299, Email: [qihuizhou@uor.edu.cn](mailto:qihuizhou@uor.edu.cn).


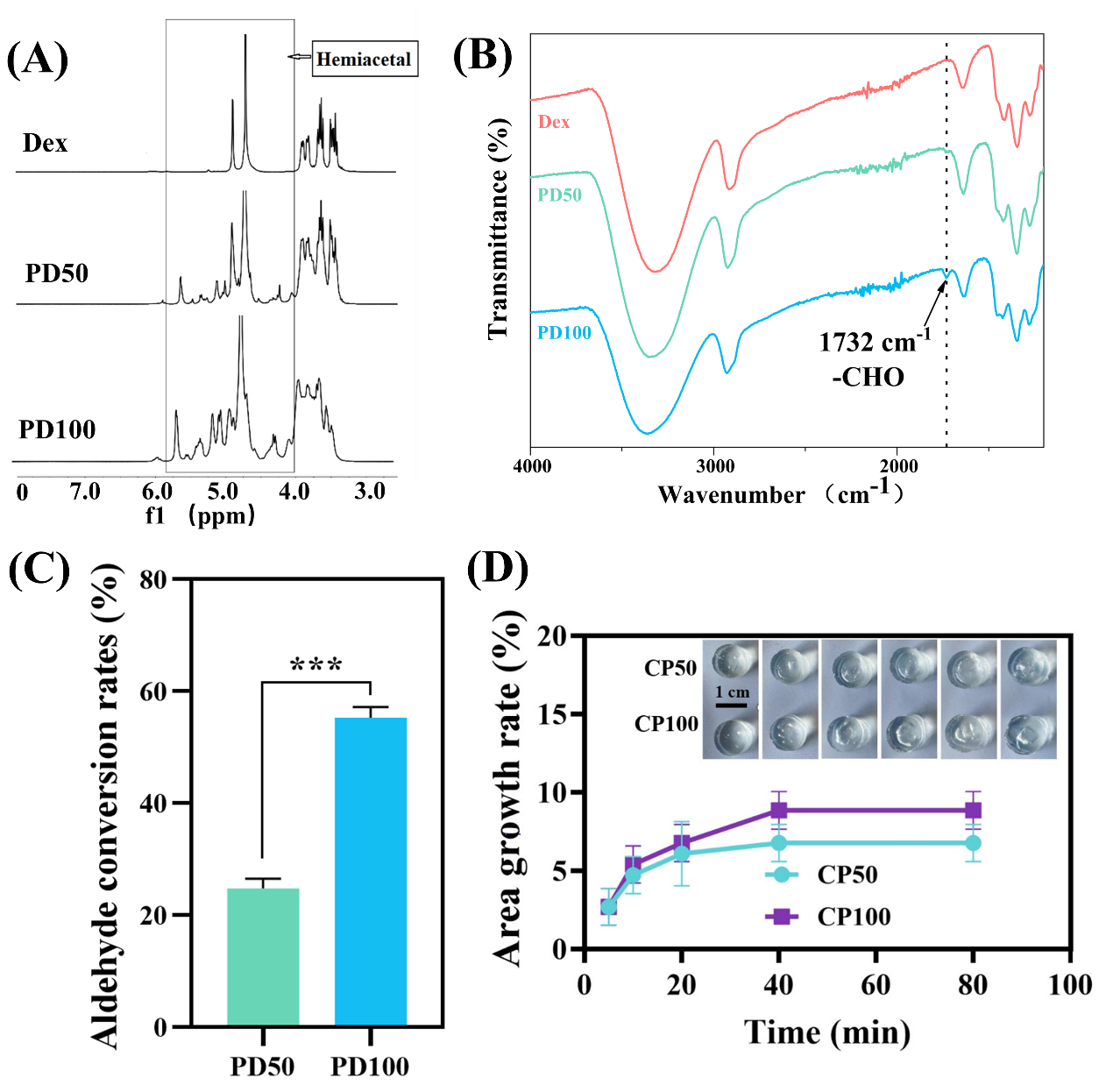


**Figure S1**. (A) ^1^H NMR spectrometer (Bruker Advance III, German) of PD0, PD50, and PD100. Compared to Dex, hemiacetal peaks at 4.0–5.6 ppm appeared in CP50 and CP100 spectra, indicating that hemiacetal groups were generated in the prepared polymer backbone. (B)Fourier transforms infrared (FTIR) spectroscopy of Dex, PD50, and PD100. The FTIR spectra of PD50 and PD100 presented a weak peak at 1732 cm^-1^ belonging to the stretching of the carbonyl from an aldehyde group, confirming the successful oxidation of dextran residues. (C) The aldehyde content of the dextran was evaluated by the hydroxylamine hydrochloride titration method (n=3). The aldehyde conversion rates of PD50 and PD100 were 24.77 ± 1.43% and 55.25 ± 1.57%, respectively. (D) After 5 minutes, all the CMCS/PD hydrogels showed an similar increase in surface area growth ratio (2.69%). Until to 80 min, a significant change in surface area growth ratio was observed, in which the area growth ratio of CP100 is 8.86% whereas that of CP50 was 6.78%.


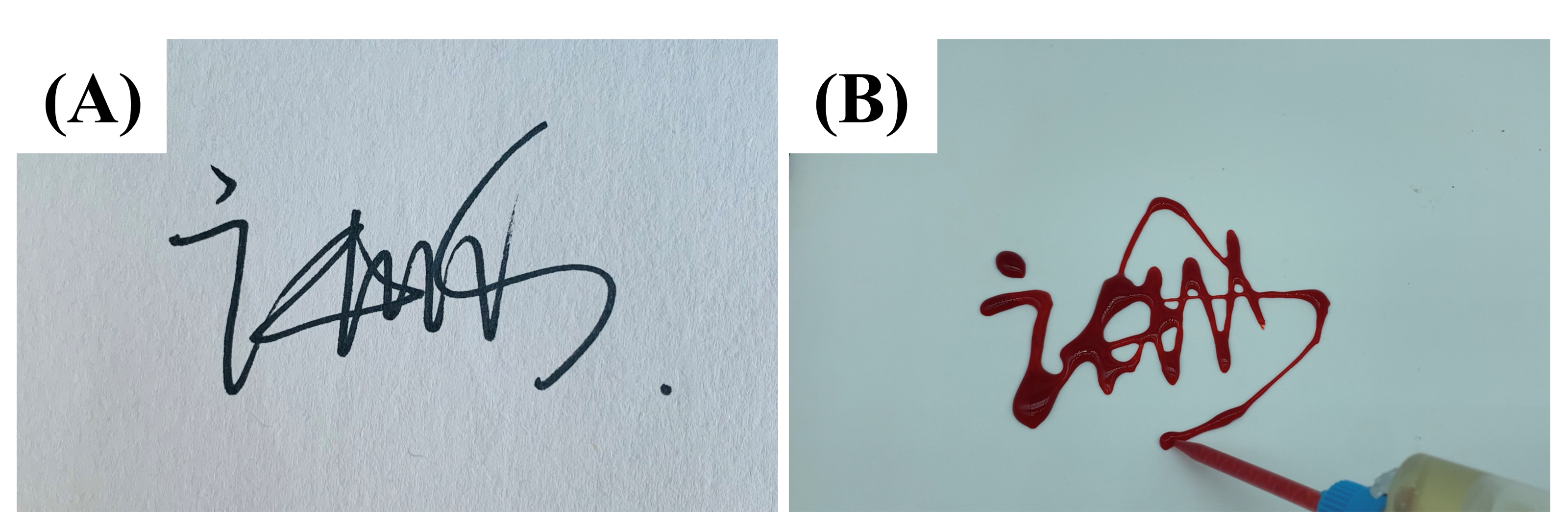


**Figure S2**. Signature by pen (A) and CP100 hydrogels (B). The macroscopic injectability of the hydrogels is shown below, which proved that the CP100 hydrogels displayed favorable injectability and rapid gelation ability. Which is well suitable for the development of injective hydrogels.


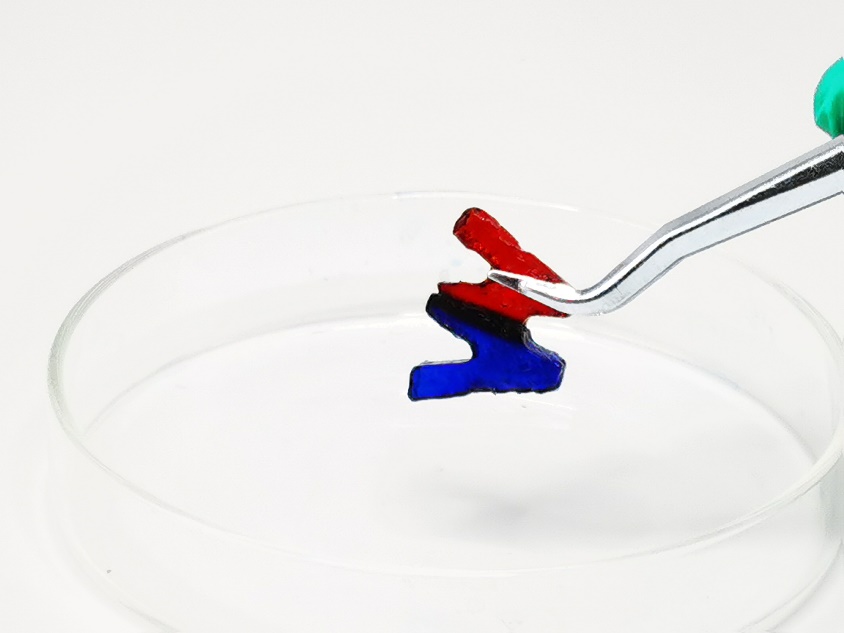


**Figure S3**. Self-healing effect of CP100. The excellent self-healing status was observed at the spliced site of hydrogels, and the formed hydrogels could effectively maintain their integrity under gravity without any external intervention.


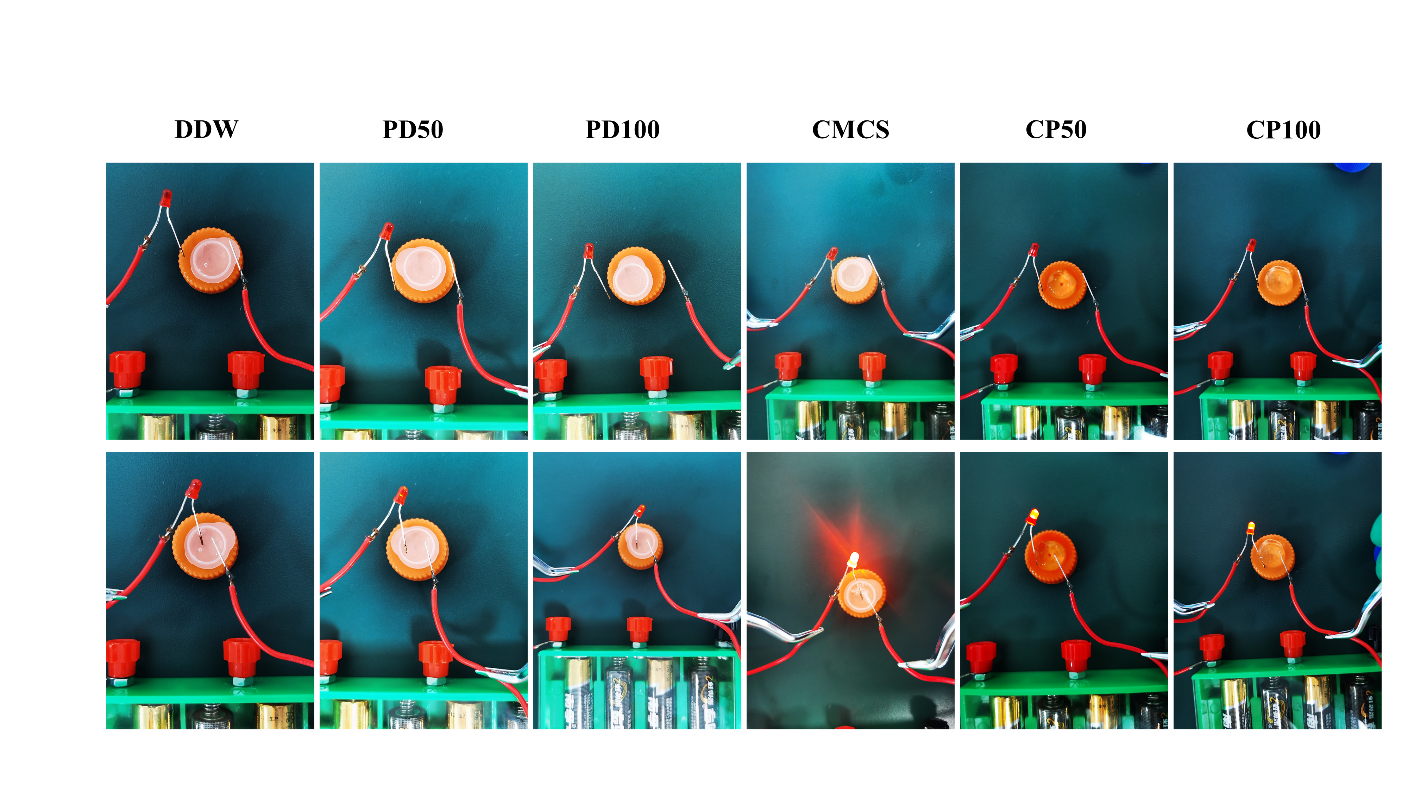


**Figure S4.** The electrical conductivity of DDW, PD50, PD100, CMCS, CP50 and CP100. CP100 showed the obvious electrical conductivity that provides prospects for application in bio-signal detection and healthcare monitoring.


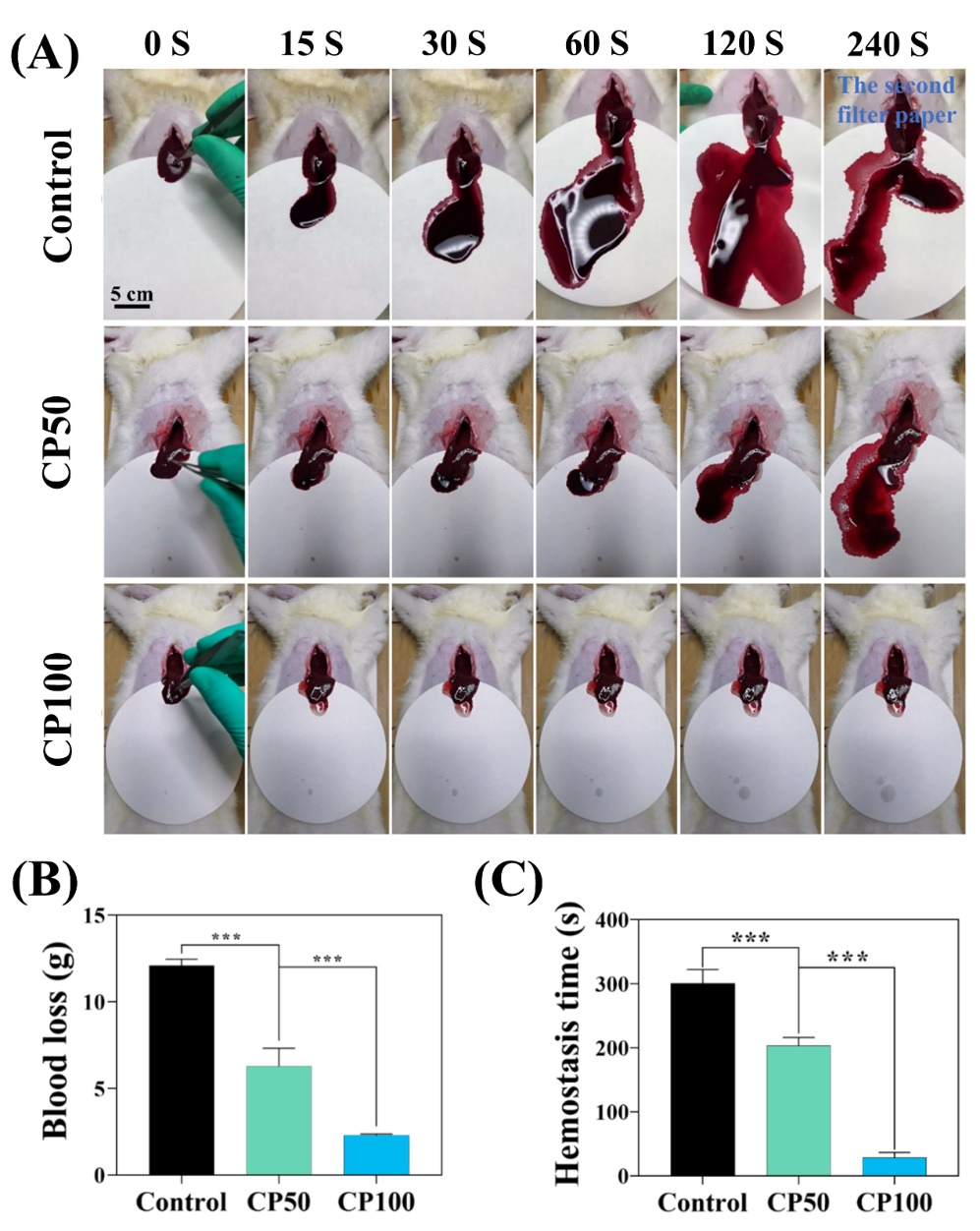


**Figure S5.** (A) Optimal images to observe the hemostatic effect of CMCS/PD hydrogels on rabbit liver bleeding model. (B-C) The quantitative blood loss and hemostasis time from image (A). Given the blood pressure of rabbits similar to humans, the cardiac hemostasis test was also performed in rabbits, and the results further clarified the excellent hemostatic effect of CMCS/PD hydrogels.
